# Current Evidence on Vitamin D Deficiency and Metabolic Syndrome in Obese Children: What Does the Evidence from Saudi Arabia Tell Us?

**DOI:** 10.3390/children5010011

**Published:** 2018-01-15

**Authors:** Asma M. Alaklabi, Naser A. Alsharairi

**Affiliations:** 1 Public Health, School of Medicine, Griffith University, Gold Coast Campus, Southport, QLD 4222, Australia; asma-188@hotmail.com; 2 Understanding Chronic Conditions, Heart, Mind & Body Research Group, Menzies Health Institute Queensland, Griffith University, Gold Coast Campus, Southport, QLD 4222, Australia

**Keywords:** children, obesity, metabolic syndrome, vitamin D, Saudi Arabia

## Abstract

Obesity and vitamin D deficiency represent major health problems among Saudi children, and have been linked to chronic diseases. Obese children are at risk of developing vitamin D deficiency, which appears to have negative influences on energy homeostasis, impeded bone mineralisation, insulin resistance and inflammation. Evidence supporting the association between vitamin D deficiency of obese children and metabolic syndrome has not specifically been studied in early childhood. The mechanisms through which vitamin D deficiency is associated with metabolic syndrome in obese children needs further elucidation. This commentary aims to (i) summarise current knowledge of the association between vitamin D deficiency and metabolic syndrome in obese children; and (ii) discuss current evidence for the association among Saudi Arabian children.

## 1. Introduction

Vitamin D deficiency among children has become a major health problem worldwide [1]. Previous research findings have indicated that vitamin D deficiency is common among children in Western countries [2,3,4,5]. Some studies examining vitamin D deficiency in Saudi Arabia have demonstrated a high prevalence of vitamin D deficiency among children and adolescents [6,7]. Low vitamin D levels can have several causes, such as abnormal intestinal function or malabsorption, reduced intake or increased degradation of vitamin D [1].

Obesity and being overweight represent major health problems among children in Saudi Arabia [8,9,10]. Vitamin D deficiency was associated with obesity, but the degree of this linkage is unclear [11,12]. Vitamin D deficiency was associated with excess body weight due to vitamin D malabsorption or the sequestration of the vitamin in adipose tissue, which may therefore affect the bioavailability of fat-soluble vitamin D in obese individuals [13]. Studies have repeatedly shown that obesity is associated with vitamin D deficiency in children and adolescents [12,14,15,16,17]. Some studies have found that vitamin D deficiency was associated with obesity among Saudi adolescents [18,19]. However, the causality of the relationship between vitamin D deficiency and obesity in children remains uncertain.

Metabolic syndrome is a clinical condition that consists of several cardiometabolic risk factors such as obesity, hypertension, dyslipidemia, and insulin resistance that occur in obese children [20,21]. Previous research has found evidence for an association between metabolic syndrome and cardiovascular risk factors (impaired fasting glucose, low-density lipoprotein (LDL) cholesterol, hypertriglyceridemia) among Saudi children and adolescents [22].

Research suggests that vitamin D deficiency has been linked to metabolic syndrome [23,24]. The empirical evidence on the association between vitamin D deficiency and metabolic syndrome in obese children is inadequate and no research has been done focusing on early childhood. In addition, the mechanism through which vitamin D deficiency is associated with metabolic syndrome in obese children has not been fully elucidated. Therefore, providing evidence from studies examining whether vitamin D deficiency is associated with metabolic syndrome in obese children is crucial. The most commonly used criteria for defining metabolic syndrome in this paper require that dyslipidemia, abnormal glucose metabolism, accelerated atherosclerosis, impaired fasting glycaemia, increased homeostatic model assessment-Insulin resistance (HOMA-IR) levels, increased systolic blood pressure and low plasma adiponectin levels be present in obese children. This commentary aims to summarise current knowledge regarding the association between vitamin D deficiency and metabolic syndrome in obese children, and to discuss current evidence for the association among Saudi Arabian children.

## 2. Vitamin D in Children: Assessment of Status and Deficiency Risk Factors

Vitamin D is a fat-soluble vitamin that distributed into serum, muscle, liver and fat tissue [25]. Vitamin D is obtained from dietary sources or made in the skin when it's exposed to sun [26,27]. There are two important forms of total body vitamin D stores; vitamin D2 (ergocalciferol) and D3 (cholecalciferol). Vitamin D2 is produced via ultraviolet B (UVB) irradiated yeast and used as a dietary supplement, whereas vitamin D3 is produced in the skin in response to exposure to the sun’s UV rays or obtained from dietary sources, supplements or fortified foods. Both forms are converted into 25(OH)D (calcidiol) in the liver, and then converted to the 1,25(OH)_2_D (calcitriol) in the kidneys which is the most active hormonal form of vitamin D [28].

Habitual intake of vitamin D in children can be estimated through the use of different tools, which differ depending on study resources, designs and objectives. The short food frequency questionnaire was found to be a valid and reliable tool to estimate vitamin D intake in children aged 6–14 years compared with other methods because it is inexpensive to administer and easy to use and analyse [29]. Other research has delivered similar findings, indicating that the short food frequency questionnaire was a valid tool to assess vitamin D in young children aged 5–7 years [30,31]. Furthermore, the assessment of vitamin D in clinical settings depends on measuring serum 25(OH)D (the summation of 25(OH)D_3_ plus 25(OH)D_2_). The best approach to assess an individual’s vitamin D status is the vitamin D Standardization Program (VDSP). A standardised laboratory measurement of 25(OH)D is the most accurate laboratory procedure to the values obtained using reference measurement procedures measured by the National Institute of Standards and Technology (NIST) and Ghent University reference measurement systems (RMPs). The main steps to achieve standardisation are (1) develop RMS; (2) establish a traceability chain from the true 25(OH)D concentration as measured by an RMP to the research laboratory; and (3) establish and verify “end-user” test performance to develop consistency across different assays [32].

In common with most population subgroups of children, including adolescents, children aged 6–16 years were at risk of developing vitamin D deficiency [33,34,35,36]. There are potential confounding factors affecting vitamin D status, which are also known to play a role in increased risk of metabolic syndrome in children. For example, increased use of TV, video or computers [37,38,39], less physical activity [33,38,39,40,41,42,43], low sun exposure [33,41,42,43,44], low levels of milk consumption [33,37,43,45] and high levels of soft drink consumption [45] can increase the risk of developing vitamin D deficiency in children aged 1–18 years. At age 6–16 years, children were vitamin D deficient in spring and winter [35,36,39,41,46,47,48] and girls were found to be more vitamin D deficient than boys [33,34,36,37,48,49,50]. Low vitamin D status is a significant concern in children aged 3–18 years from some racial/ethnic minorities and urban communities in USA, Central and Western Europe, Southern Asia and Eastern Africa [39,40,41,44,45,47,48,50].

## 3. Vitamin D Deficiency and Metabolic Syndrome Risk Factors in Obese Children

Poor vitamin D levels appear to have a negative influence on energy homeostasis and insulin resistance in obese adolescents. The mechanism by which vitamin D influences these conditions in obese adolescents is poorly understood. The underlying process could involve insulin secretion by pancreatic β cells or attenuation of inflammation [51] or sequestration and altered metabolism in adipose tissue [11,12]. Low vitamin D levels lead to decreased bioavailability of vitamin D in obese adults due to its sequestration in body fat [13,52]. Recent evidence has revealed that vitamin D deficiency is prevalent among obese adults, and vitamin D stores were greater in obese adults than in non-obese adults [53]. Available data suggests that vitamin D3 is present in fat tissue in larger amounts than vitamin D2 [13]. Research has found that the blood concentration of vitamin D3 was higher in obese than in non-obese adults following exposure to solar UVB. The obese adults produced more vitamin D3 due to their larger body surface available to exposure than non-obese adults [52]. There is little evidence to support the hypothesis that fat tissue and serum vitamin D concentrations are positively correlated in obese adults. The positive association is due to the fact that fat tissue is the storage site for vitamin D3. It is perhaps notable that obese adults produced enough vitamin D to raise the circulating of vitamin D3 by increasing their exposure to sunlight [54].

The etiology of metabolic syndrome is complex and can be influenced by a number of contributing factors. Metabolic syndrome is influenced by many factors such as obesity, smoking, alcohol intake, increasing age, unhealthy diet and lack of exercise [55,56,57,58]. Vitamin D deficiency can be considered as a risk factor for metabolic syndrome [59]. Previous cross-sectional studies suggest that low vitamin D levels have been linked to hypertension, obesity, high density lipoprotein, systolic blood pressure, fasting blood glucose, insulin resistance, hyperglycemia and dyslipidaemia in children [42,60,61,62,63,64,65] and adolescents [66,67,68,69,70].

A growing body of epidemiological studies suggests an association between vitamin D deficiency and obesity in children and adolescents [12,14,15,38,41,44,47,48]. However, the causality of the association has been uncertain. Low 25(OH)D levels may work as a marker rather than work as a primary influence on obesity [28]. Given that the predominant storage site for vitamin D is adipose tissue, it is most likely that the high storage capacity of vitamin D in obese adults leads to low 25(OH)D levels [52]. Observational studies found it difficult to prove that obesity causes vitamin D deficiency because low 25(OH)D levels might be attributed to lifestyle, socioeconomic or dietary factors (referred to as confounding variables). There are also no randomized controlled trial (RCT) studies specifically designed to assess the relationship between vitamin D deficiency and obesity in children. However, in a Mendelian randomisation meta-analysis in adults, the causality of the association between obesity and vitamin D deficiency is explored using a genetic variants instrument. It was stated that if low 25(OH)D levels lead to obesity, then genetic variants associated with low 25(OH)D levels should be linked to high body mass index (BMI). On the other hand, if obesity is causally associated with low 25(OH)D levels, then genetic variants associated with high BMI should be linked to low 25(OH)D levels [71]. Data from non-interventional observational trials have demonstrated no causal relationship between vitamin D deficiency and metabolic syndrome such as type 2 diabetes mellitus in children [72]. On the other hand, a mendelian randomisation analysis that combined case-control and cross-sectional datasets confirms a causal relationship between obesity and cardiometabolic risk factors (i.e., type 2 diabetes, blood pressure, blood lipids, glycemic phenotypes) in adults [73]. Therefore, it is possible that it is obesity and not vitamin D deficiency that causes metabolic syndrome in children.

Obese children were at greater risk of vitamin D deficiency and metabolic risk factors than normal weight children [15]. Previous cross-sectional studies found that low serum 25(OH)D level was associated with abnormal glucose metabolism and increased HOMA-IR levels [74,75,76,77,78], dyslipidemia [65], impaired fasting glycaemia [74,75,76,77,78,79,80], low plasma adiponectin levels [75,76,77], increased systolic blood pressure [74], decreased high-density lipoprotein-cholesterol [77,78,79,80,81] and accelerated atherosclerosis [82] in obese children aged 5–20 years when compared to normal weight children. No other studies have considered an association between vitamin D deficiency and metabolic syndrome in obese children prospectively.

## 4. Current Evidence in Saudi Arabia

The prevalence of obesity and overweight among children is high. Across Saudi Arabia, Central, Eastern and Northern regions reported the highest rates of obesity among children and adolescents [83,84,85]. The prevalence of obesity and overweight among children (aged 2–18 years) in the Eastern province is 19% and 23.3% respectively [86]. A national survey in 2005 reported that the prevalence of obesity and overweight among children aged 5–18 years are 10.1% and 22.4% in boys and 8.4% and 23.8% in girls respectively [87]. A recent cross-sectional data survey conducted in Riyadh, found that the prevalence of overweight and obesity among children aged 2–14 years is 9.5% and 13.5% in boys and 14.4% and 18% in girls respectively [88]. The high prevalence of childhood obesity may result in serious health implications; diabetes mellitus, hypertension and dyslipidemia are most common in Saudi children, and can contribute to the development of metabolic syndrome [89].

Metabolic syndrome among children has become a serious health problem in the Arab Gulf countries [90,91,92]. It has been reported that the prevalence of metabolic syndrome among Saudi children and adolescents aged 10–18 years was 10.3% for boys and 8.1% for girls [93]. The high prevalence of metabolic syndrome among Saudi children and adolescents is attributed to increasing obesity, diabetes mellitus, dyslipidemia and high LDL cholesterol [93,94]. Metabolic syndrome is also attributed to physical inactivity and high levels of consumption of energy dense foods among children. Children and adolescents consume snack and fast foods and low fruit, vegetable, milk and breakfast. They were also physically inactive and spent time in sedentary behaviours such as TV watching [95,96,97,98]. The dietary intakes of adolescents aged 13–18 years typically include foods containing low levels of micronutrients such as calcium, phosphorus, potassium, vitamin D, vitamin E, selenium and manganese [99].

Vitamin D deficiency is a major health problem among Saudi Arabian children [6,7]. Evidence has demonstrated that children aged 4–18 years with vitamin D deficiency had low exposure to sunlight and were physically inactive [100,101]. Epidemiological evidence investigating the association between vitamin D status and metabolic risk factors has come from studies on adults. A study examined vitamin D deficiency associated with coronary heart disease among 130 adults (aged 19–49 years). Vitamin D deficiency was used as having 25(OH)D < 20 ng/mL. The study found that vitamin D deficiency was associated with coronary heart disease. Results also showed that vitamin D deficiency was more common in coronary heart disease cases than in controls [102]. A recent case-control study aimed to investigate whether attitudes, behaviours and knowledge related to vitamin D contribute to the increased prevalence of vitamin D deficiency in adults aged ≥19 years. The following cut-off of 25(OH)D was used to define vitamin D status: deficiency 25(OH)D < 20 ng/mL; insufficiency 10 to < 19.9 ng/mL; sufficiency ≥20 ng/mL; severe vitamin D deficiency < 10 ng/mL. The study found that vitamin D deficiency was associated with low levels of knowledge about vitamin D and low intake of vitamin supplements [103]. One study found that vitamin D deficiency (25(OH)D < 37.5 nmol/L) was associated with insulin resistance in obese adults aged 18–25 years [104]. To date, only limited examination has been made of the associations between the vitamin D status of obese children and metabolic risk factors in Saudi Arabia. The only evidence to date, to our knowledge, to have used a case control study to evaluate vitamin D status and its association with metabolic risk factors was undertaken among 120 obese and 120 non-obese children (aged 9–14 years) at King Saud Medical city. The available evidence suggests that serum 25(OH)D was positively associated with high density lipoprotein cholesterol and negatively associated with triglyceride, LDL cholesterol, fasting blood glucose and BMI. Results also showed that obese children had worse metabolic status than non-obese children [105]. Since there is limited evidence about evaluating vitamin D’s effectiveness in preventing cardiovascular diseases in the field of childhood nutrition research in Saudi Arabia, a more comprehensive investigation of the association between vitamin D status and metabolic risk factors in obese children would be required. A better understanding of the complexity of the relationship between vitamin D status and metabolic risk factors in obese children could enable researchers to develop appropriate health promotion schemes and future interventions targeting children, aimed at increasing knowledge and awareness about the benefits of vitamin D and potentially reducing the risk of obesity and metabolic syndrome in Saudi Arabia.

## 5. Conclusions

Current cross-sectional evidence suggests that vitamin D deficiency is associated with metabolic syndrome risk factors in obese children. No evidence to date has sought to discover if there is an association in early childhood. The prevalence of vitamin D deficiency among children was found to be dependent on confounding factors such as ethnicity, sex, low physical activity, low sun exposure, increased TV watching, low milk consumption and high soft drink consumption, which are also considered to play a role in increased risk of metabolic syndrome. Prospective studies are warranted to establish whether there is a causal association between vitamin D levels and metabolic syndrome in obese children.

The prevalence of obesity has increased in Saudi Arabia at an alarming rate; as a consequence, the risk factors for developing metabolic syndrome are found to be high among children. Although obesity and metabolic syndrome risk factors are well recognised as health problems in Saudi Arabia, limited studies have been undertaken, and health strategies have been largely ignored. The mechanisms that potentially link vitamin D deficiency with metabolic syndrome risk factors in obese children are so far poorly documented and need to be elucidated through future studies, which might provide health strategies aimed at reducing the risk of obesity and metabolic syndrome among children in Saudi Arabia.
